# The role of the CX3CL1/CX3CR1 axis as potential inflammatory biomarkers in subjects with periodontitis and rheumatoid arthritis: A systematic review

**DOI:** 10.1002/iid3.1181

**Published:** 2024-02-06

**Authors:** Mario A. Alarcón‐Sánchez, Julieta S. Becerra‐Ruiz, Celia Guerrero‐Velázquez, Seyed A. Mosaddad, Artak Heboyan

**Affiliations:** ^1^ Biomedical Science, Faculty of Chemical‐Biological Sciences Autonomous University of Guerrero Guerrero Mexico; ^2^ Institute of Research of Bioscience, University Center of Los Altos University of Guadalajara Guadalajara Mexico; ^3^ Research Center in Molecular Biology of Chronic Diseases, Southern University Center University of Guadalajara Guadalajara Mexico; ^4^ Department of Research Analytics, Saveetha Dental College and Hospitals, Saveetha Institute of Medical and Technical Sciences Saveetha University Chennai India; ^5^ Student Research Committee, School of Dentistry Shiraz University of Medical Sciences Shiraz Iran; ^6^ Department of Prosthodontics, Faculty of Stomatology Yerevan State Medical University after Mkhitar Heratsi Yerevan Armenia; ^7^ Department of Prosthodontics Tehran University of Medical Sciences Tehran Iran

**Keywords:** biomarkers of inflammation, chemokines, CX3CL1, CX3CR1, fractalkine, periodontitis, rheumatoid arthritis

## Abstract

**Objective:**

This systematic review aimed to investigate the role of the C‐X3‐C motif ligand 1/chemokine receptor 1 C‐X3‐C motif (CX3CL1/CX3CR1) axis in the pathogenesis of periodontitis. Furthermore, as a secondary objective, we determine whether the CX3CL1/CX3CR1 axis could be considered complementary to clinical parameters to distinguish between periodontitis and rheumatoid arthritis (RA) and/or systemically healthy subjects.

**Methods:**

The protocol used for this review was registered in OSF (10.17605/OSF.IO/KU8FJ). This study was designed following Preferred Reporting Items for Systematic Review and Meta‐Analysis guidelines. Records were identified using different search engines (PubMed/MEDLINE, Scopus, Science Direct, and Web of Science) from August 10, 2006, to September 15, 2023. The observational studies on human subjects diagnosed with periodontitis and RA and/or systemically healthy were selected to analyze CX3CL1 and CX3CR1 biomarkers. The methodological validity of the selected articles was assessed using NIH.

**Results:**

Six articles were included. Biological samples (gingival crevicular fluid [GCF], saliva, gingival tissue biopsies, serum) from 379 subjects (*n* = 275 exposure group and *n* = 104 control group) were analyzed. Higher CX3CL1 and CX3CR1 chemokine levels were found in subjects with periodontitis and RA compared with periodontal and systemically healthy subjects.

**Conclusion:**

Very few studies highlight the role of the CX3CL1/CX3CR1 axis in the pathogenesis of periodontitis; however, increased levels of these chemokines are observed in different biological samples (GCF, gingival tissue, saliva, and serum) from subjects with periodontitis and RA compared with their healthy controls. Future studies should focus on long‐term follow‐up of subjects and monitoring changes in cytokine levels before and after periodontal therapy to deduce an appropriate interval in health and disease conditions.

## Graphical abstract

This systematic review aimed to investigate the role of the Fractalkin‐CX3CL1/CX3CR1 axis as pro‐inflammatory biomarkers to distinguish between periodontitis and rheumatoid arthritis and/or systemically healthy subjects.

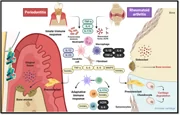

## 1 INTRODUCTION

Periodontitis is a chronic and multifactorial inflammatory disease caused by constant exposure to a polymicrobial dysbiotic film with a high prevalence of Gram‐negative bacteria such as *Prevotella intermedia*, *Fusobacterium nucleatum*, *Aggregatibacter actinomycetemcomitans*, *Porphyromonas gingivalis*, *Tannerella forsythia*, and *Treponema denticola*, and in the presence of a dysregulated immune response in a genetically susceptible host.^1^ Periodontitis is considered the sixth most common osteolytic disease affecting humans,^2^ has a prevalence of 62.3%, and in its most severe form, can affect up to 23.6% of the world population.^3^ The most current classification proposed by the global workshop is based on the complexity/severity and progression of the disease represented by Stages I–IV and Grades A–C.^4^ Affected individuals clinically present bleeding on probing (BOP), suppuration, dental mobility, changes in the levels of insertion (clinical attachment loss [CAL]), and bone loss, ultimately leading to extraction or loss of teeth, which has a significant impact on the individual's oral health‐related quality of life.^5^ Therefore, its diagnosis is by clinical and radiographic examination. Treatment consists of oral biofilm control through patient education, personalized oral hygiene instructions, and the removal of supra‐ and subgingival dental plaque by scaling and root planing, and administering antimicrobial agents placed directly into the periodontal pockets.^1^, ^4^

Rheumatoid arthritis (RA) is a chronic autoimmune disease characterized by inflammation and destruction of cartilage and bone in the joints with subsequent functional limitation.^6^ Patients show swelling, deformity, and stiffness as specific disease symptoms.^7^ It affects up to 1% of the world population. Its etiology is related to the presence of environmental (socioeconomic status, smoking, and diet) and genetic factors, as well as dysbiosis of the oral and intestinal microbiome (infections) resulting in the synthesis of autoantibodies such as anticitrullinated protein antibodies (ACPAs).^8^ Some periodontopathogenic bacteria, such as *P. gingivalis* and *A. actinomycetemcomitans*, have been associated with increased citrullination.^9^ In addition, these pathogens can induce other key immunological mechanisms such as the formation of neutrophil extracellular traps (NETs) through the induction of NETosis, can also stimulate pro‐inflammatory responses by the T helper‐17 subset of T cells through activation of the interleukin‐23/IL‐17 (IL‐23/IL‐17) axis and can promote osteoclastogenesis, which ultimately leads to bone damage and systemic inflammation.^10^ Therefore, a bidirectional causal relationship between periodontitis and RA has been established.^11^

**Figure 1:**
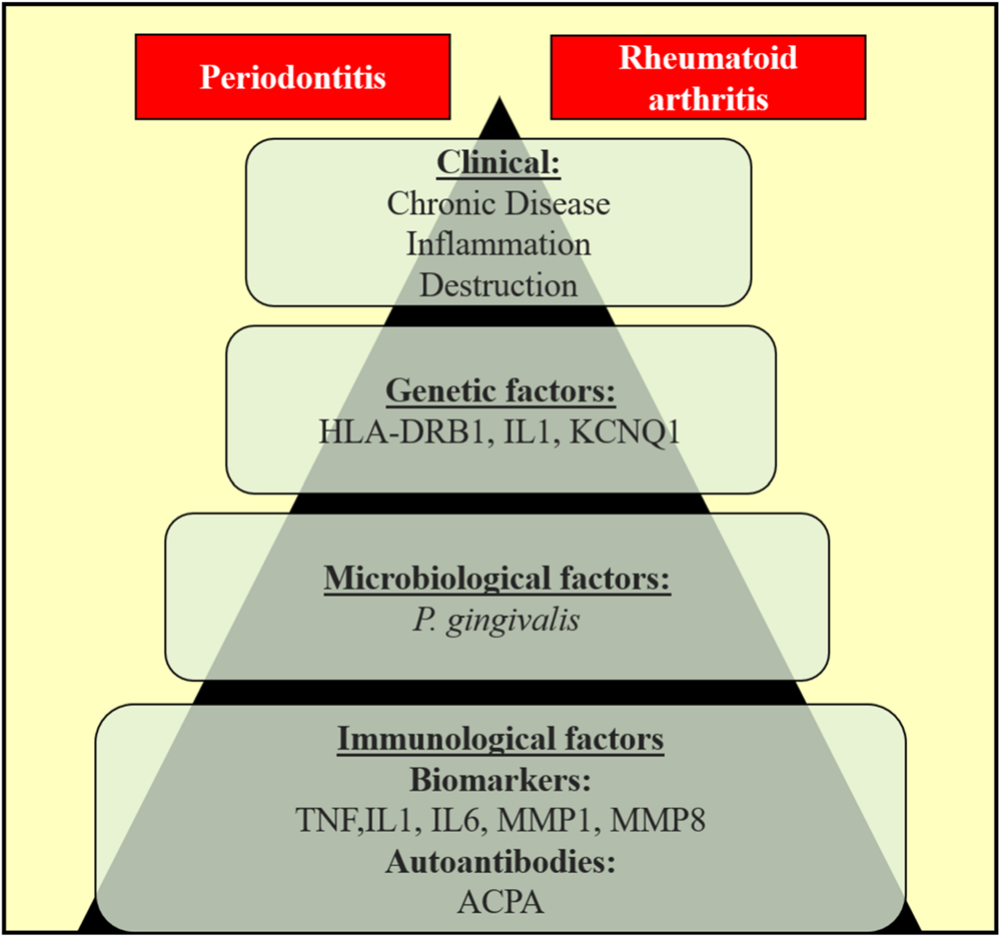
Clinical, genetic, microbiological, and immunological characteristics that subjects with periodontitis and rheumatoid arthritis (RA) share with each other.^12^, ^13^ Both diseases are chronic and characterized by the presence of inflammation and destruction of the previously mentioned tissues. Genetically, the highly polymorphic HLA‐DRB1 (shared epitope, SE) locus represents the most strongly involved genetic factor related to susceptibility and severity in developing both diseases. Concerning microbiological factors, clinical studies indicate that subjects with periodontitis and *Porphyromonas gingivalis* infection are at risk of developing RA. In addition, in subjects with periodontitis and/or RA, increased levels of pro‐inflammatory cytokines (TNF, IL‐1, IL‐6, MMP) and antibodies such as ACPA have been observed. Abbreviations: ACPA, anti‐citrullinated protein antibodies; HLA‐DRB1, human leukocyte antigen; IL1, interleukin‐1; IL‐6 Interleukin‐6; KCNQ1, cardiac potassium channels; MMPs, matrix metalloproteinases; TNF, tumor necrosis factor.

Scientific evidence has shown that both diseases are associated and have clinical, genetic, and microbiological characteristics in common.^12^, ^13^ Additionally, immunopathogenic similarities are present, as shown in Figure 1.^14^

**Figure 2:**
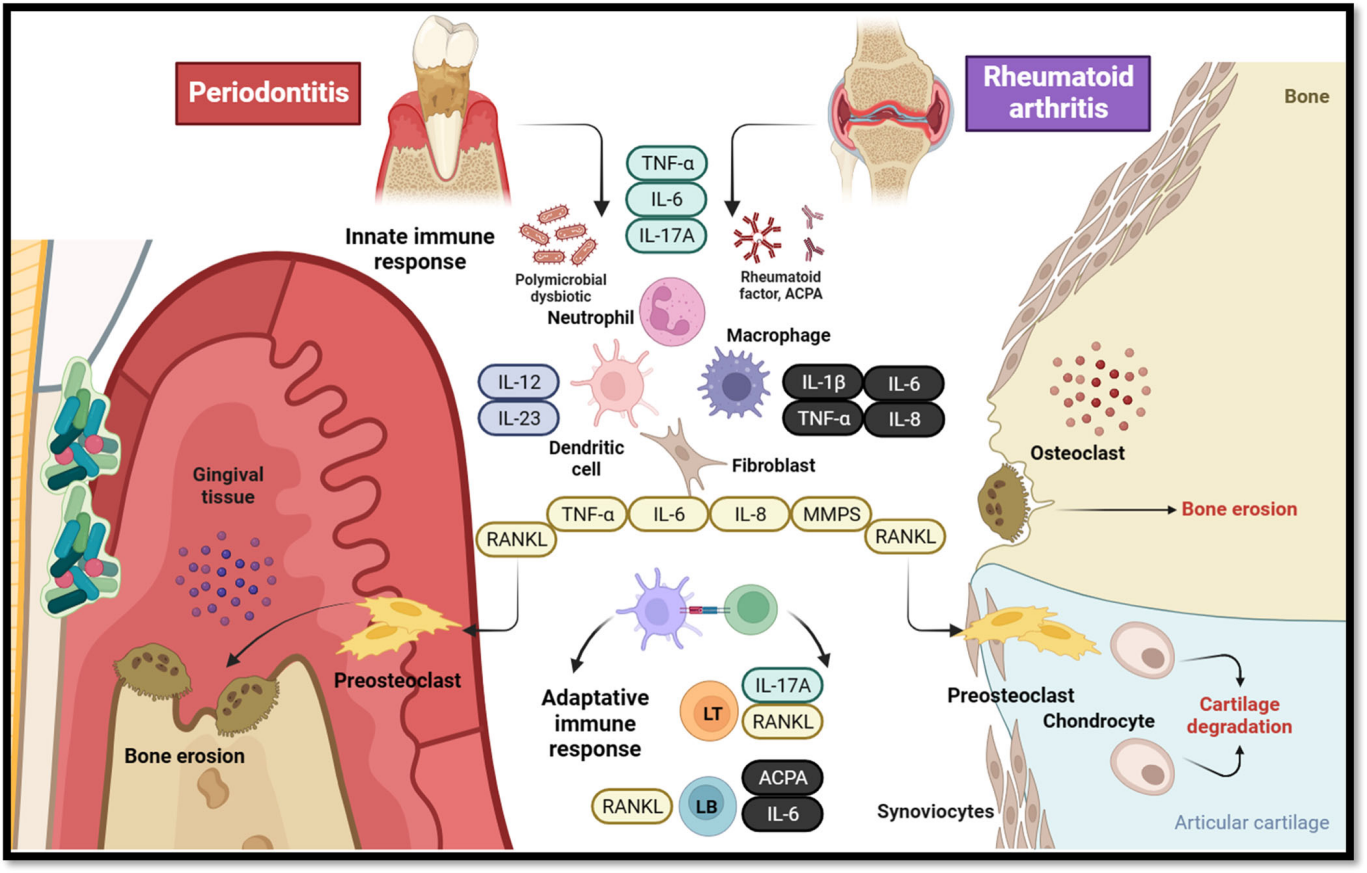
Immunological interactions between periodontitis and rheumatoid arthritis. IL, interleukin; MMPS, matrix metalloproteinases; RANKL, receptor activator of nuclear factor κ‐Β ligand; TNF‐α, tumor necrosis factor‐α.

In response to a dysbiotic periodontal microbiome, which adheres to the acquired salivary film that coats the surface of teeth, implants, or dentures (as occurs in periodontitis) and/or to the formation of ACPA and rheumatoid factor (RF) (as occurs in RA), the epithelial cells, fibroblasts, and innate immune cells (neutrophils, macrophages, dendritic cells) increase the production both locally and systemically of cytokine/chemokine levels such as tumor necrosis factor‐α (TNF‐α), IL‐6, IL‐17, IL‐12, IL‐23, IL‐1β, IL‐8, and C‐X3‐C motif ligand 1 (CX3CL1) which perpetuate the pro‐inflammatory state due to the formation of a positive feedback loop. Eventually, this leads to the destruction of the supporting tissues of the teeth and joints^15^ (Figure 2). However, what is the role of chemokines in both diseases?

Chemokines are low molecular‐weight proteins involved in biological processes such as hematopoiesis, angiogenesis, degranulation, and chemotaxis, particularly in immune‐mediated diseases.^16^ To date, 50 members (ligands) have been distinguished, which are classified into four subfamilies according to the number and spacing of conserved cysteine residues at the N‐terminal end: subfamily XC, CC, CXC, and CX3C.^17^ At the same time, the 19 chemokine receptors are coupled to heterotrimeric G proteins^18^ and are named XCR, CCR, CXCR, and CX3CR, respectively.^19^

A chemokine of special interest is CX3CL1 or fractalkine (FKN),^20^ which was discovered more than 25 years ago as the only member of the CX3C chemokine subclass, which can be synthesized as a type I transmembrane protein, with a molecular weight of 100 kDa and consisting of 375 amino acids, or in its soluble form with a molecular weight of 85 kDa and consisting of 317 amino acids. FKN has a dual function by interacting with and binding to its receptor, the chemokine receptor 1 C‐X3‐C motif (CX3CR1), either as a cell adhesion molecule or a potent immune cell chemoattractant.^21^ Thus, the CX3CL1/CX3CR1 axis is implicated in several important diseases such as atherosclerosis,^22^ chronic kidney disease,^23^ diffuse parenchymal diseases,^24^ cancer, Alzheimer's disease,^25^ and human immunodeficiency virus (HIV).^26^ The role of CX3CL1/CX3CR1 in RA is already documented and can be consulted in other reviews.^17^, ^20^, ^27^, ^28^ Meanwhile, its relationship with periodontitis has recently been demonstrated by evaluating its expression in different biological samples as a possible inflammatory biomarker.^29^, ^30^, ^31^, ^32^, ^33^, ^34^ In this context, CX3CL1 expression has been shown to have different functions in both diseases. On the one hand, it promotes the migration of monocytes, T cells, and osteoclast precursors to both periodontal and synovial tissues. It also induces the production of pro‐inflammatory mediators (TNF‐α, IL‐6, and IL‐1) by macrophages, T cells, gingival fibroblasts, and synoviocytes. An essential aspect concerning bone pathobiology is that it promotes osteoclastogenesis.^12^, ^13^, ^14^

A comprehensive knowledge of the role of the CX3CL1/CX3CR1 axis could contribute to the understanding of the pathogenesis of both diseases; likewise, its expression pattern may be helpful in the determination of parameters and new therapeutic strategies, acting as a possible therapeutic target for the resolution of any of the forms of disease.

Thus, the present systematic review aimed:

- To investigate the role of the CX3CL1/CX3CR1 axis in the pathogenesis of periodontitis.
- To determine whether the CX3CL1/CX3CR1 axis could be considered as a complementary tool to clinical parameters to distinguish between periodontitis and RA and/or systemically healthy subjects.

## 2 MATERIALS AND METHODS

### 2.1 Protocol development

The present systematic review was performed according to the Preferred Reporting Items for Systematic Review and Meta‐Analysisnguidelines.^35^ The protocol was recorded with the OSF enrollment (Registration DOI. 10.17605/OSF.IO/KU8FJ).

## 3 PICO

The PICO strategy was used to formulate the research question.

– P (population): Subjects with periodontitis.
– I (intervention): Role of CX3CL1 and CX3CR1 chemokines.
– C (comparison): Subjects with RA and/or systemically healthy.
– O (outcome): Not applicable.

### 3.1 Review question

What is the role of the CX3CL1/CX3CR1 axis in subjects with periodontitis?

With the subquestion: Could the CX3CL1/CX3CR1 axis be used as a complementary tool to clinical parameters to distinguish between periodontitis and RA and/or systemically healthy subjects?

### 3.2 Eligibility criteria

The inclusion criteria were as follows:

- Articles published after 2000.
- Articles published in peer‐reviewed and indexed journals in Journal Citation Reports.
- Original research: clinical, cross‐sectional, retrospective, or prospective studies analyzing the chemokines CX3CL1 and/or CX3CR1 in periodontitis and RA, or systemically healthy subjects.
- Studies with quantification of CX3CL1 and/or CX3CR1 chemokines in gingival crevicular fluid (GCF), gingival tissue biopsies, saliva, or blood serum.
- Absence of antibiotics, anti‐inflammatory drugs, other comorbidities, and periodontal treatment in the inclusion criteria.
- Articles in English.

The exclusion criteria were as follows:

- Articles published before 2000.
- Non‐peer‐reviewed/non‐indexed journals.
- Narrative, scoping, systematic reviews, and meta‐analyses were excluded. Letters to the editor and brief communications were also excluded.
- Those studies where CX3CL1 and/or CX3CR1 levels were analyzed in subjects with periodontitis and/or RA, together with other comorbidities and/or interventions (drug treatments).
- Edentulous patients and patients with orthodontic appliances.
- Articles in a language other than English.

### 3.3 Search strategy

A digital search was performed in PubMed/MEDLINE, Scopus, Science Direct, and Web of Science databases. The search was conducted from August 10, 2006, to September 15, 2023, with no time restrictions on published articles. However, the search was limited to clinical studies. For the PubMed library, the search terms were as follows: (periodontal disease [MeSH Terms]) OR periodontitis [MeSH] Terms]) OR gingivitis [Title/Abstract]) OR inflammatory biomarkers [Title/Abstract]) OR gingival crevicular fluid [Title/Abstract]) OR GCF [Title/Abstract]) OR rheumatoid arthritis [Title/Abstract]) OR fractalkine [Title/Abstract]) OR CX3CL1 [Title/Abstract]) AND (cytokines [MeSH Terms]) OR chemokines [Title/Abstract]) OR cytokines [Title/Abstract]). For Scopus, the search strategy was: (“periodontitis”/AND “rheumatoid arthritis”/AND “CX3CL1”/AND “CX3CR1”). We applied (chemokine OR inflammatory biomarker) AND periodontitis for Science Direct. For Web of Science, we used (cytokine OR biomarker) AND periodontitis AND rheumatoid arthritis. For the journals: “*Journal of Periodontal Research*,” “*Journal of Periodontal and Implant Science*,” “*Journal of Periodontology*,” “*Journal of Clinical Periodontology*” and “*Periodontology 2000*” manual searches were performed.

### 3.4 Study selection and data extraction

Considering the eligibility criteria, two investigators independently (M.A.A.S. and J.S.B.R.) reviewed the articles by reading the titles and abstracts from the search results. They then summarized each article's content, assessed the studies’ validity, and identified duplications. The same investigators selected articles that met the inclusion criteria or those with insufficient data. Any disagreement was resolved by discussion with a third investigator (A.H). Subsequently, data extraction was performed independently from the articles selected for qualitative analysis. A comprehensive analysis of the data was performed. For each study, the information collected was as follows:

- Name of first author and year of publication.
- Country where the research was conducted.
- Title and objectives of the study.
- Journal where the article was published.
- Study design.
- Registration by the ethics committee.
- The institution where the research was carried out.
- Inclusion and exclusion criteria.
- Classification of periodontitis and RA.
- Periodontal clinical parameters.
- Gender and age.
- Sample size.
- Type of statistical analysis.
- Type of biological samples.
- Type of biomarkers evaluated.
- Type of assay/kits used.
- Main findings and conclusions.

### 3.5 Quality assessment of included studies

Methodological quality and risk of bias were independently assessed by two investigators (M.A.A.S. and C.G.V.) based on the NIH Quality Assessment Tool for Observational Cohort and Cross‐Sectional Studies 26 criteria, which consisted of 12 questions assessing the research question, study population, exposure, outcomes, follow‐up rate, and statistical analysis. Thus, the studies were rated as fair and good.^36^

## 4 RESULTS

### 4.1 Selection of studies

**Figure 3:**
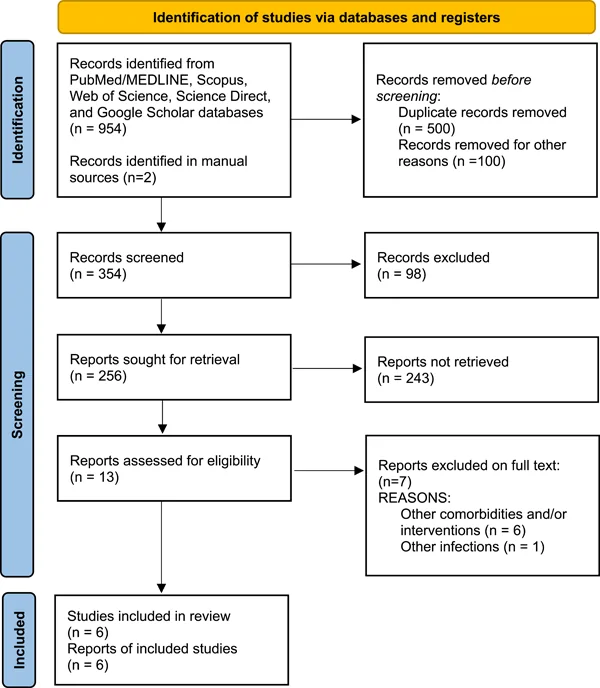
PRISMA flow diagram. PRISMA, Preferred Reporting Items for Systematic and Meta‐Analyses.

Initially, the literature search of the electronic databases yielded 954 publications (Figure 3). A manual search and review of all bibliographies yielded an additional 956 results. Of these, 600 records were removed before screening. Subsequently, 256 articles remained after the exclusion of duplicates. After screening, 13 articles were reviewed and classified as potentially relevant. As a result, seven articles whose focus differed from the research question were excluded. Six were excluded because they included patients with RA and other conditions and/or interventions that would modify the response variable. One more was excluded because the study population had periapical lesions. Finally, six studies were analyzed for this systematic review. Table 1 lists these studies’ main characteristics regarding study design, structure, and conduct. Some studies differed in terms of statistical heterogeneity:

**Table 1:** Overview of the included studies.

Study characteristics	Author/year/reference					
	Balci et al., 2021^32^	Yilmaz et al., 2020^29^	Kawamoto et al., 2020^33^	Panezai et al., 2020^30^	Panezai et al., 2017^31^	Hosokawa et al., 2005^34^
Country	Turkey	Turkey	Brazil	Pakistan	Pakistan	Japan
Article title	Potential biomarkers reflecting inflammation in patients with severe periodontitis: Fractalkine (CX3CL1) and its receptor (CX3CR1)	Salivary and serum concentrations of monocyte chemoattractant protein‐1, macrophage inhibitory factor, and Fractalkine in relation to RA and periodontitis	Chemokines and cytokines profile in whole saliva of patients with periodontitis.	Upregulation of circulation inflammatory biomarkers under the influence of periodontal disease in rheumatoid arthritis patients	Correlation of serum cytokines, chemokines, growth factors, and enzymes with periodontal disease parameters	Expression of Fractalkine (CX3CL1) and its receptor, CX3CR1, in periodontal diseased tissue
Objective	To determine differences in GCF and serum levels of CX3CL1/CX3CR1 between the patients with Stage III/Grade B periodontitis and periodontally healthy subjects	To examine the effects of periodontitis and RA on their serum and salivary concentrations	To analyze chemokines/cytokines profile in whole saliva of individuals with severe periodontitis (Stage III) presenting moderate (Grade B; GB) or rapid progression rate with a localized incisor‐molar ‐pattern (Grade C; GC/IMP)	To assess the systemic inflammation and disease activity of RA under the influence of PD	To investigate serum cytokines, chemokines, growth factors, enzymes, and costimulatory proteins in association with periodontal conditions in PD and RA subjects	To analyze the expression of CX3CL1 and CX3CR1 by RT‐PCR and immunohistochemistry in gingival tissues. In addition, CX3CL1 production in LPS‐stimulated endothelial cells was evaluated by ELISA
Journal of publication	*Journal of Periodontal Research*	*Journal of Periodontology*	*Cytokine*	*Cytokine*	*PLOS ONE*	*Clinical and Experimental Immunology*
Study design	Cross‐sectional	Cross‐sectional	Cross‐sectional	Cross‐sectional	Cross‐sectional	Cross‐sectional
Ethics register	Yes	Yes	Yes	Yes	Yes	Yes
Institution	Istanbul Medipol University	Sakarya University, Faculty of Medicine	Biomedical Science Institute of University of São Paulo	Altamash Institute of Dental Medicine in Karachi	Altamash Institute of Dental Medicine in Karachi	Tokushima University
Inclusion criteria	Patients with Stage III Grade B periodontitis	Patients with RA and Stage III Grade B periodontitis	Patients with Stage III Grade B and Stage III Grade C with a localized incisor‐molar‐pattern	Patients with RA and chronic periodontitis	Patients with RA and chronic periodontitis	Patients with chronic periodontitis and systemically healthy
Exclusion criteria	Having <20 teeth, using tobacco products, systemic disease (diabetes mellitus and RA), being pregnant or lactating, antibiotics or nonsteroid anti‐inflammatory drugs used in the last 6 months, received periodontal therapy other than scaling and polishing in the previous 6 months	Having <16 teeth, reporting the intake of antibiotics and/or having received periodontal treatment during the preceding 6 months before the initiation of the study, having a chronic disease or related medication use with no effect on the periodontium, having been diagnosed with other forms of RA, being pregnant or in the lactating period, carrying genetic renal and hepatic disorders or HIV, or having a history of transplantation	Previously periodontal therapy in the last 6 months, use of medications that could affect the periodontium, such as corticosteroids or antibiotics in the previous 6 months, and/or mouthwashes containing antimicrobials, systemic diseases that could affect the progression of the periodontitis, pregnant or lactating, smokers	A history of periodontal treatment <6 months and/or treatment with antibiotics in < 3 months	Individuals with osteoarthritis, gout, and a history of treatment for PD during the last 6 months and/or treatment with antibiotics in the previous 3 months	Systemic antibiotic, anti‐inflammatory, hormonal, or other assisted drug therapy in the 6 months before the study. Patients who had received previous periodontal treatment in the last 2 years
Periodontal classification	2017 Classification of Periodontal and Peri‐Implant Diseases and Conditions	2017 Classification of Periodontal and Peri‐Implant Diseases and Conditions	2017 Classification of Periodontal and Peri‐Implant Diseases and Conditions	Not available	Not available	American Academy of Periodontology
RA classification	Not available	American College of Rhematology	Not available	ACR/EULAR	ACR/EULAR	Not available
Investigation parameters	PI, PPD, GR, CAL, BOP	PI, GI, PPD, CAL, BOP	BOP, PPD, CAL	BOP, PPD	BOP, PPD	PPD, CAL
Gender	^a^25(62.5)	^b^89(100)	^a^21(65.3)	^a^59(77.6)	^a^64(71)	^a^25(62.5)
^a^F/^b^M	^b^15(48.5)		^b^11(34.7)	^b^17(22.4)	^b^26(29)	^b^15(48.5)
Average age	33.7 ± 8.59	52.25 ± 0.1	32.34 ± 4.32	46.56 ± 10.96	46.4 ± 10.1	33.7 ± 8.59
Cases	*n* = 20	*n* = 65	*n* = 16	*n* = 76	*n* = 76	*n* = 20
	P/SIII/GB = 20	RA + P/SIII/GB = 23	P/SIII/GB = 9	RA + P = 19	RA + P = 19	P = 20
		RA = 23	P/SIII/GC = 7	RA = 19	RA = 19	
		P/SIII/GB = 21		P = 38	P = 38	
Controls	20	22	16	12	14	20
Total study population	40	89	32	88	90	40
Statistical analysis	Shapiro–Wilk test, Wilcoxon, Mann–Whitney *U* test, Student's, and Spearman's correlation	Kolmogorov–Smirnov test, Kruskal–Wallis, *χ* ^2^ test, and Spearman's correlation	Kolmogorov–Smirnov, Mann–Whitney *U* test, Benjamini–Hochburg multiple testing corrections, Multivariate analysis, Permutational multivariate analysis of variance	Shapiro–Wilk test, Student *t* test and one‐way analysis of variance with Tukey–Kramer posthoc test, Mann–Whitney *U* test, Kruskall–Wallis, Spearman's correlation	Spearman's correlation	Student's *t* test
Biological sample	GCF Serum	Saliva Serum	Saliva	Serum	Serum	Gingival tissue
Biomarkers	CX3CL1 CX3CR1	CX3CL1	CX3CL1 CX3CR1	CX3CL1	CX3CL1	CX3CL1 CX3CR1
Type of assay/kit	ELISA kits (ELISA Cloud Immunoassay, Cloud‐Clone Corp)	Flow cytometry‐based technique	Multiplex assay instrument (Luminex Bio‐plex®200 Array System, Bio‐Rad)	Proximity extension assay (Olink Bioscience)	Proximity extension assay (Olink Bioscience)	RT‐PCR, immunohistochemistry, ELISA (R&D Systems)
Main findings	CX3CL1 in GCF:	CX3CL1 levels in the saliva of RA + P patients were higher compared with RA, P, and C patients, whereas in serum, CX3CL1 levels were higher in RA patients compared with RA + P, P, and C patients	CX3CL1/CX3CR1 levels were higher in patients with Stage III/Grade C periodontitis compared with their control group	CX3CL1 levels were increased in patients with RA + P compared with RA patients without P and those with P and systemically healthy	A negative (inverse) correlation exists between CX3CL1 levels and clinical parameters: BOP, PPD >5mm, and MBL	CX3CL1 and CX3CR1 mRNA was detected in five tissues of seven samples with periodontitis, whereas no mRNA was detected in normal gingival tissues
	P = 11.19 ± 2.1 pg/mL					
	C = 9.47 ± 1.4 pg/mL					
	Serum:					
	P = 112.29 ± 31.6 pg/mL					
						CX3CL1 was mainly distributed in endothelial cells; furthermore, a higher amount of infiltrating CX3CR1 expressing cells was detected in tissue with periodontitis compared to healthy tissue
	C = 143.83 ± 77.32 pg/mL					
	CX3CR1 in GCF:					
	P = 13.83 ± 7.32 pg/mL					
	C = 9.82 ± 2.25 pg/mL					
	Serum:					
	P = 1.11 ± 0.42 pg/mL					Finally, CX3CL1 expression in HUVEC cells was upregulated by LPS from *P. gingivalis*.
	C = 1.55 ± 0.64 pg/mL					
Conclusion	There was an increase in the levels of CX3CL1 and its receptor CX3CR1 in GCF from patients with Stage III/Grade B periodontitis compared with periodontally healthy patients. Meanwhile, in serum, inverse levels of these chemokines were found	Increased salivary concentrations of this chemokine may be related to altered macrophage activation, a shared factor in the pathogenesis of RA and periodontitis	These preliminary data revealed that each periodontitis phenotype had distinct immune profiles differentially expressed in saliva compared to their related controls, suggesting differences in the etiopathogenesis of GB and GC/localized incisor‐molar	Periodontitis increases systemic inflammation in RA. There is a profound influence independent of autoimmune status	Systemic inflammatory burden, via known and novel markers, is associated with periodontal conditions in PD and RA patients. Shallow pockets are not associated with a higher inflammatory state	The CX3CL1/CX3CR1 system may play an essential role in the infiltration of leukocytes into PD tissue, which might contribute to the progression of periodontitis
	Researching this novel chemokine to understand better its role in PD pathogenesis is required to establish the role of FKN and its receptor in PD and its clinical consequences					

Abbreviations: ACR/EULAR, American College of Rheumatology/European League against Rheumatism; BOP, bleeding on probing; CAL, clinical attachment loss; CX3CL1, chemokine (C‐X3‐C motif) ligand 1; CX3CR1, CX3C motif chemokine receptor 1; ELISA, enzyme‐linked immunosorbent assay; FKN, fractalkine; GCF, gingival crevicular fluid; GI, gingival index; GR, gingival retraction; HIV, human immunodeficiency virus; HUVEC, human umbilical vein endothelial cell; LPS, lipopolysaccharide; MBL, marginal bone loss; mRNA, messenger RNA; P, periodontitis; PD, periodontal disease; PI, plaque index; PPD, periodontal probing depth; RA, rheumatoid arthritis; RT‐PCR, reverse transcription polymerase chain reaction.

#### 4.1.1 Methodological heterogeneity

- From *n* = 90 to *n* = 32, significantly different numbers of cases.
- Three studies analyze CX3CL1/CX3CR1 axis levels in periodontitis subjects without RA. Although the other three studies analyze subjects with both conditions (P + RA).
- When measuring the results, only one study describes the actual levels of chemokines. The rest only do graphical descriptions of actual values.

#### 4.1.2 Clinical heterogeneity

- Variability of participants, intervention characteristics, and type of outcome measurements.
- Minimal ethnic distribution; studies only from Turkey, Brazil, Pakistan, and Japan.
- Mean age of subjects: 30–50 years old.
- The type of periodontitis not clearly classified.
- Different clinical parameters evaluated.
- Assay types vary widely.
- CX3CR1 not assessed in RA patients.

### 4.2 Risk of bias

Table 2 shows the risk of bias analysis of the studies included in this systematic review according to NIH for observational cohort and cross‐sectional studies. All articles defined Items #1–5, 9, and 12. No article met Items#8, 10, and 11. Articles^29^, ^30^, ^31^, ^33^, ^34^ did not meet Item#7, whereas articles^31^, ^34^ did not meet Item#6. Thus, most included articles met at least 80% of the evaluated items.

**Table 2:** Quality assessment of the included studies according to the NIH Quality Assessment Tool for Observational Cohort and Cross‐Selectional studies.

Questions ¿?		Author/year/reference					
		Balci et al., 2021^32^	Yilmaz et al., 2020^29^	Kawamoto et al., 2020^33^	Panezai et al., 2020^30^	Panezai et al., 2017^31^	Hosokawa et al., 2005^34^
1	Research question	Yes	Yes	Yes	Yes	Yes	Yes
2	Study population	Yes	Yes	Yes	Yes	Yes	Yes
3	Participant rate of eligible persons	Yes	Yes	Yes	Yes	Yes	Yes
4	Eligibility criteria	Yes	Yes	Yes	Yes	Yes	Yes
5	Sample size	Yes	Yes	Yes	Yes	Yes	Yes
6	Exposure assessment	Yes	Yes	Yes	Yes	No	No
7	Exposure levels	Yes	No	No	No	No	No
8	Repeated exposure assessment	No	No	No	No	No	No
9	Outcomes measures	Yes	Yes	Yes	Yes	Yes	Yes
10	Assessors blinding	No	No	No	No	No	No
11	Follow‐up rate	No	No	No	No	No	No
12	Statistical analyses	Yes	Yes	Yes	Yes	Yes	Yes
	Total	9	8	8	8	7	8

### 4.3 Characteristics of the included studies

The six (100%) articles under discussion were observational studies published between 2005 and 2021, and all met the requirements for approval by the Institutional Ethics Committee.^29^, ^30^, ^31^, ^32^, ^33^, ^34^ Of the included studies, 33.3% were conducted in Turkey^29^, ^31^ and in Pakistan,^30^, ^31^ and 17% in Brazil^33^ and in Japan.^34^ According to the available data, a total of 379 (100%) subjects participated in the six studies; among them, 194 (51%) were women and 185 (49%) were men, with an age range between 32 and 52 years. On the other hand, 104 (27.4%) were periodontal and systemically healthy subjects (control groups), 153 (40.3%) had periodontitis and were systemically healthy, 61 (16%) had RA and were periodontally healthy, and another 61 (16%) had RA and periodontitis. Of the subjects with periodontitis, only three (50%) studies defined the type of periodontitis according to the new 2017 classification of periodontal and peri‐implant diseases,^29^, ^32^, ^33^ one study (17%) defined the type of periodontitis according to the American Academy of Periodontology,^34^, ^37^ whereas the rest (33.3%) did not specify the type of periodontitis, only describing it as chronic.^30^, ^31^ Of the RA subjects, two (67%) studies used the classification by the American College of Rheumatology/European League against Rheumatism,^30^, ^31^, ^38^ whereas the other study (33.3%) used the American College of Rheumatology classification.^29^ Only one study (17%) excluded smoking subjects.^33^ Meanwhile, all six studies (100%)^29^, ^30^, ^31^, ^32^, ^33^, ^34^ excluded subjects under treatment with antibiotics and/or anti‐inflammatory drugs at the time of the study or 6 months before the study, as well as subjects who had undergone periodontal treatment. Regarding clinical parameters, all six (100%) studies^29^, ^30^, ^31^, ^32^, ^33^, ^34^ evaluated periodontal disease (PD), five (83.3%) studies evaluated BOP,^29^, ^30^, ^31^, ^32^, ^33^ four (67%) evaluated CAL,^29^, ^32^, ^33^, ^34^ and only two (33.3%) plaque index.^29^, ^32^

### 4.4 Results of CX3CL1/CX3CR1 levels in different biological samples in periodontitis and RA subjects, and/or systemically healthy subjects

Four studies (67%) evaluated chemokine levels in serum samples,^29^, ^30^, ^31^, ^32^ two studies (33.3%) did so in saliva,^29^, ^33^ one study (17%) in GCF,^32^ and one in gingival tissue.^32^ Balci et al.^32^ found a significant increase in CX3CL1, CX3CR1, and IL‐1β levels in GCF of subjects with stage III/grade B periodontitis compared to periodontally healthy subjects. However, when analyzing serum samples, they found inverse levels, that is, increased levels of these chemokines and cytokines in periodontally healthy subjects compared with periodontitis subjects. Also, they found positive correlations between the levels of inflammatory markers in GCF and clinical parameters. Yilmaz et al.^29^ found a significant increase in CX3CL1 levels in the saliva of RA subjects compared with Stage III–IV/Grade B periodontitis subjects with RA, periodontitis subjects, and systemically healthy and control subjects. In serum, CX3CL1 levels were higher in subjects with RA and Stage III/Grade B periodontitis compared to the other groups. Compared with the previous study, this study demonstrated elevated levels of this chemokine in the serum of subjects with periodontitis compared with their controls. In addition, clinical parameters were higher in RA + P subjects than in periodontitis subjects and systemically healthy subjects. Kawamoto et al.^33^ found increased levels of CX3CL1/CX3CR1 in the saliva of subjects with Stage III/Grade B and C periodontitis compared with their controls; however, these results were insignificant. Panezai et al.^30^ found a significant increase in serum CX3CL1 levels in subjects with RA and chronic periodontitis compared to the other groups (RA, P, and control). Furthermore, they found positive correlations between the levels of this chemokine and different rheumatological parameters (Rheumatoid Arthritis Disease Activity Index DAS‐28, RF, ACPA, and erythrocyte sedimentation rate). Panezai et al.^31^ unlike Balci,^32^ found negative correlations between CX3CL1 levels and clinical parameters such as BOP, periodontal probing depth, and marginal bone loss. Finally, Hosokawa et al.^34^ were the first to describe the role of the CX3CL1/CX3CR1 axis in subjects with periodontitis. First, they found an increased expression of CX3CL1 messenger RNA (mRNA) in gingival tissue biopsies. Furthermore, immunohistochemistry showed a strong expression of the axis in endothelial cells and CD3+ T cells in inflamed tissues of subjects with periodontitis. They also demonstrated that human umbilical vein endothelial cells exposed to lipopolysaccharide (LPS) from *Escherichia coli*, *P. gingivalis*, lipoteichoic acid from *Staphylococcus aureus*, and TNF‐α increase the production of CX3CL1.

## 5 DISCUSSION

This systematic review aimed to investigate the role of the CX3CL1/CX3CR1 axis in the pathogenesis of periodontitis and RA. Furthermore, our secondary objective was to determine whether the CX3CL1/CX3CR1 axis could be considered a clinical tool complementary to clinical parameters to distinguish between periodontitis and RA and/or systemically healthy subjects. Thus far, the CX3CL1/CX3CR1 axis's role in periodontitis is largely unsupported by the literature, with only six published studies.^29^, ^30^, ^31^, ^32^, ^33^, ^34^ However, in other diseases such as cancer, renal disease, neurological and cardiovascular disorders, HIV, and RA, its role has already been investigated.^17^, ^20^, ^22^, ^23^, ^24^, ^25^, ^26^, ^27^, ^28^, ^33^ The two objectives of this review will be discussed below.

### 5.1 Structure of CX3CL1/CX3CR1 axis

FKN (CX3CL1) or neurotactin is a chemokine first characterized by Bazan et al. in the late 1990s. The gene encoding is located on chromosome 16q13 and is the only chemokine in the CX3C subfamily, Category δ (dual function).^39^ Structurally, it is characterized by separating three amino acid residues at its N‐terminal cysteine motif. CX3CL1 is generally produced by neurons, synoviocytes, endothelial cells, smooth muscle cells, and monocytes; however, keratinocytes, osteoblasts, and gingival fibroblasts also synthesize it.^40^

Once the mRNA encoding this protein is translated, it undergoes glycosylation processes. It is then incorporated into the plasma membrane as a transmembrane protein (mCX3CL1), whose primary function is to act as a cell adhesion molecule, facilitating further accumulation and retention of leukocytes at the injury site.^41^ On the other hand, it can also present as a soluble protein (sCX3CL1); this molecular form of the protein is produced by proteolysis due to the participation of different enzymes such as TNF‐α‐converting enzyme/disintegrin‐like metalloproteinase 17 (ADAM17), disintegrin domain‐containing protein and metalloproteinase 10 (ADAM 10), matrix metalloproteinase 3 (MMP‐3) and cathepsin S that cleave the transmembrane domain giving rise to a potent chemoattractant for leukocytes. Both molecular forms of the protein can be induced by pro‐inflammatory cytokines such as IL‐1β, TNF‐α, interferon‐γ (IFN‐γ), as well as virulence factors such as LPS from bacteria like *P. gingivalis* and even oxygen tension.^14^, ^16^, ^17^, ^42^

The CX3CR1 is the only member of the heterotrimeric G‐protein‐coupled transmembrane receptor superfamily of receptors. It is primarily expressed in monocytes/macrophages, osteoclasts, gingival fibroblasts, natural killer cells, and cytotoxic T lymphocytes.^41^ The gene encoding the CX3CR1 protein is located on chromosome 3p22.2 and was identified 1 year after the discovery of its ligand by Combadiére et al. in 1998.^43^ Structurally, the protein contains seven transmembrane domains (α‐helical) that penetrate the thickness of the plasma membrane. In addition to its binding to CX3CL1, it can also interact with another chemokine, such as CC chemokine ligand type 2, although its affinity is 10–20 times lower. Finally, the interaction and binding of this receptor leads to the activation of multiple signaling pathways, such as MAPK/ERK, PI3K/AKT/PKB, FAK, and JAK2/STAT3.^44^, ^45^

### 5.2 Direct and indirect role of CX3CL1/CX3CR1 axis in the inflammatory environment

The CX3CL1/CX3CR1 axis, as well as the interaction with other chemokines, can act from a homeostatic context, which explains the trafficking and patrolling of basal leukocytes through the vascular endothelium and the architecture of secondary lymphoid organs. As well as from an inflammatory context through the recruitment of leukocytes to the target tissue in the presence of trauma, pathologies, or infections that could harm the individual.^46^ In the latter scenario, the interaction between FKN and its receptor enhances transient capture and binding of leukocytes to endothelial cells, which is followed by crawling/firm adhesion (activation of integrins by chemokines), production of pro‐inflammatory cytokines and transmigration through the endothelial layer to sites of inflammation.^17^

### 5.3 Uncovering the role of the CX3CL1/CX3CR1 axis in periodontitis

Physiologically, a healthy periodontium can maintain an excellent dynamic balance between cytokines and chemokines to control inflammation. Destruction of periodontal tissue occurs when this balance is disrupted and shifts in favor of increased production of pro‐inflammatory cytokines and chemokines by immune cells.^47^ Periodontitis‐related pro‐inflammatory cytokines, such as TNF‐α and IL‐1β produced from host cells exposed to dental biofilm, produce a synergistic effect by increasing the synthesis of IL‐6, IL‐8, prostaglandin‐E_2_, MMPs, such MMP‐8, MMP‐9, and MMP‐13, whose primary substrate is collagen type I and III present in the alveolar bone, as well as CX3CL1, which perpetuates the inflammatory state and results in further destruction of periodontal tissues.^48^, ^49^

The onset and progression of periodontitis are attributed to essential bacteria; in particular, severe forms of periodontitis are caused by *A. actinomycetemcomitans*, *F. nucleatum*, *P. gingivalis*, *T. forsythia*, and *T. denticola* present in higher proportion in dental biofilm which stimulate the inflammatory immune response, resulting in tissue damage in susceptible individuals.^6^ In humans with periodontitis, there is an increased amount of CX3CL1.^29^, ^30^, ^31^, ^33^, ^34^ Virulence factors released by periodontopathogenic bacteria cause the production of this chemokine by gingival keratinocytes and fibroblasts. In humans, CX3CL1 causes the recruitment of neutrophils and monocytes that become macrophages at inflamed gingival sites. Once neutrophils arrive at gingival tissues and in the presence of critical bacteria or pathobionts, they polarize towards the N1 phenotype that secrete cytokines that allow macrophage differentiation towards an M1 phenotype that promotes microbiota dysbiosis, inflammation, and alveolar bone loss. CX3CL1 also acts on osteoclast precursor cells that migrate through the osteoblast layer.^50^, ^51^, ^52^ Thus, CX3CL1 is a potent chemoattractant for preosteoclasts and causes osteoclastogenesis, destroying periodontal tissues.^53^

Regarding pharmacological agents targeting the CX3CL1/CX3CR1 axis in the pathogenesis of periodontitis, it has been shown that the therapeutic CX3CR1 antagonist called “F1” was able to inhibit leukocyte recruitment in a murine model of thioglycolate‐induce periodontitis.^54^, ^55^ The mechanism of action of the drugs that have been developed to date focuses on blocking CX3CR1 through direct binding to the active site or allosteric modulation with repercussions on downstream signaling pathways.^23^ Current drug therapy targeting the CX3CL1/CX3CR1 axis is summarized in Table 3.

**Table 3:** Current drug therapy targeting the CX3CL1/CX3CR1 axis.

Drug name	Company	Type	Animal models reported	Human clinical trial in progress
F1	INSERM	Modified CX3CR1 ligand	Periodontitis	N/A
BI665088	Boehringer–Ingelheim/Ablynx	VHH Antibody to CX3CR1	Atherosclerosis	Chronic kidney disease
E6011	Eisai Co	Humanized monoclonal antibody	Pharmacokinetics in cynomolgus monkeys reported	RA Inflammatory bowel disease
AZD8797	Astra Zeneca	Small molecule inhibitor	Spinal cord injury Multiple sclerosis	N/A
E6130	Eisai Co	Small molecule inhibitor	Inflammatory bowel disease	N/A
JMS 17‐2	Drexel University College of Medicine	Small molecule inhibitor	Breast cancer metastasis	N/A

Abbreviations: CXCL1, C‐X3‐C motif ligand 1; CX3CR1, chemokine receptor 1 C‐X3‐C motif; N/A, not applicable; RA, rheumatoid arthritis; VHH, variable domain of camelid, heavy chain only.^17^

### 5.4 Findings on the participation of the CX3CL1/CX3CR1 axis as potential inflammatory biomarkers in periodontitis, RA, and other oral conditions

In recent years, biomarkers of inflammation have been used as molecular tools for diagnosing periodontitis.^2^ The most frequently studied inflammatory mediators are cytokines such as TNF‐α, IFN‐γ, IL‐10, IL‐17A, IL‐6, IL‐23, IL‐21, and chemokines such as IL‐8, CXCL10, CXCL13, CX3CL1, and its receptor CX3CR1, the latter two being potentially promising markers. The primary oral fluids that facilitate the study of these biomarkers are GCF, gingival tissue biopsies, and saliva; however, blood serum samples have also contributed to their detection at the systemic level.^50^, ^56^ Thus, increased expression and levels of the FKN‐CX3CL1/CX3CR1 axis have been observed in subjects with mainly Stage III/Grade B and C periodontitis compared with their healthy controls.^29^, ^30^, ^31^ As well as in subjects with RA.^22^, ^23^, ^24^, ^45^, ^57^ It would be premature to assert, however, that the CX3CL1/CX3CR1 axis can be leveraged as a clinical tool to distinguish between the status of patients with RA and periodontitis and that of periodontal and systemically healthy individuals in the lack of clinical data. This, in addition to considering that, ideally, a biomarker should meet certain important criteria, such as validity, ease of use, affordability, and cost‐effectiveness, and should be collected in a noninvasive manner.^49^ Likewise, as an interesting finding strongly related to periodontitis, it has been shown that this axis is related to the development of periapical lesions by participating in the progression of tissue destruction, including bone resorption, during the inflammatory process of peri‐radicular tissues.^58^ On the other hand, concerning prosthetic restorations, the potential effects of dental implants (titanium) and amalgam restorations that present a long time of evolution have been evaluated with the levels of amino acids such as l‐Kyn/l‐Trp and chemokines such as MCP‐1 and CX3CL1, finding that CX3CL1 levels were higher in those subjects who had titanium implants and long‐standing dental amalgams compared with subjects with only long‐standing dental amalgams.^59^ These studies suggest that the FKN‐CX3CL1/CX3CR1 axis plays a vital role in the pathogenesis of periodontitis, as it could be associated with various mechanisms aimed at regulating the inflammatory process affecting the periodontium, especially the recruitment of specific leukocytes to the damaged area.

### 5.5 Limitations and future perspectives

Although the importance of the FKN‐CX3CL1/CX3CR1 axis in neurological and autoimmune diseases is well established, little is known about the interaction between CX3CL1 and other CX3CR1 ligands whose expression is also increased in inflammatory processes such as periodontitis.^17^ Experimental evidence in murine models or cell lines of the oral cavity is also minimal; it would be essential to know in depth the molecular mechanisms that mediate the destruction of alveolar bone and the attraction of leukocytes to the site of the inflamed periodontal tissue. In addition, this axis could be studied in subjects with periodontitis and other related systemic diseases, such as obesity, cardiovascular disease, and oral cancer, as it has been shown to have proangiogenic activities; however, it would be advisable to use a larger sample size. Also, it would be essential to know the response of the gingival tissue in the release of this chemokine in the presence of periodontopathogenic species such as *P. gingivalis*, *T. denticola*, and *T. forsythia*, which under conditions of dysbiosis and through the release of their virulence factors would favor the destruction of the tissues that support the tooth.^60^, ^61^

Finally, knowing or considering the molecular factors at the expression level of the chemokine genes and their receptors such as single nucleotide polymorphisms that can affect up or down the expression of the cytokine, and these can differ from one population to another, being in some a protective factor, whereas in others a risk factor for the development of diseases.^62^

## 6 CONCLUSION

Within the limitations of the present study and despite its promising clinical applications, it is too early to state that the CX3CL1/CX3CR1 axis can be used as a clinical tool to differentiate the condition of subjects with periodontitis and RA from periodontally and systemically healthy subjects. Very few studies highlight the role of the CX3CL1/CX3CR1 axis in the pathogenesis of periodontitis; however, increased levels of these chemokines are observed in different biological samples (GCF, gingival tissue, saliva, and serum) from subjects with periodontitis and RA compared to their healthy controls. Future studies should focus on long‐term follow‐up of subjects and monitoring changes in cytokine levels before and after periodontal therapy to deduce an appropriate interval in health and disease conditions.

## AUTHOR CONTRIBUTIONS

*Conceptualization*: Mario A. Alarcón‐Sánchez and Julieta S. Becerra‐Ruiz. *Methodology*: Mario A. Alarcón‐Sánchez and Julieta S. Becerra‐Ruiz. *Software*: Celia Guerrero‐Velázquez. *Validation*: Mario A. Alarcón‐Sánchez and Celia Guerrero‐Velázquez. *Formal analysis*: Mario A. Alarcón‐Sánchez and Celia Guerrero‐Velázquez. *Investigation*: Mario A. Alarcón‐Sánchez and Seyed A. Mosaddad. *Resources*: Mario A. Alarcón‐Sánchez. *Data curation*: Julieta S. Becerra‐Ruiz. *Writing—original draft preparation*: Mario A. Alarcón‐Sánchez. *Writing—review and editing*: Seyed A. Mosaddad and Artak Heboyan. *Visualization*: Julieta S. Becerra‐Ruiz. *Supervision*: Mario A. Alarcón‐Sánchez. *Project administration*: Artak Heboyan. All authors have read and approved the published version of the manuscript.

## CONFLICT OF INTEREST STATEMENT

The authors declare no conflict of interest.

## ETHICS STATEMENT

Not applicable.

## Data Availability

Data used in structuring this study will be available upon request.
